# Psychometric properties of the Tuckman Academic Procrastination Scale (TPS) in university students from Ecuador

**DOI:** 10.1186/s41155-025-00365-y

**Published:** 2025-11-10

**Authors:** Jessica Vanessa Quito Calle, Alejandro César Cosentino, Luis Ernesto Quito Calle, Lauro Esteban Cañizares Abril, Andrés Ramírez

**Affiliations:** 1 Carrera de Psicología Clínica, Grupo de Investigación en Neurociencia Clínica Aplicada (GINCA), Universidad Politécnica Salesiana, Cuenca, Ecuador; 2 Department of Psychology, Faculty of Social Sciences, Universidad de Palermo, Buenos Aires, Argentina; 3 Instituto San José de Calasanz, Cuenca, Ecuador

**Keywords:** Psychometric, Procrastination, Reliability

## Abstract

**Background:**

Academic procrastination, defined as the intentional delay of important tasks, is a frequent phenomenon among university students and is associated with low performance, stress, and anxiety. The Tuckman Academic Procrastination Scale (TPS) is a widely used psychometric tool to assess this behavior. Although it has been validated in several countries, there is no empirical evidence regarding its validity and reliability in the Ecuadorian context, which limits its use for assessment and intervention.

**Objective:**

To psychometrically validate the Ecuadorian version of the TPS (TPS-E) in university students, assessing its factor structure, reliability, and cultural adequacy for measuring academic procrastination.

**Methods:**

An instrumental design was used with a non-probabilistic sample of 1,007 students (39.9% women; M_age_ = 21.88; SD = 3.69) from a private Ecuadorian university. The Spanish adaptation (*Argentinean*) was linguistically reviewed and applied using a 16-item Likert-type scale. Confirmatory Factor Analysis (CFA) with the DWLS estimator on a polychoric correlation matrix was conducted, evaluating CFI, RMSEA, and SRMR indices. Internal consistency was estimated using Cronbach’s alpha and McDonald’s omega.

**Results:**

The initial unidimensional model (16 items) showed acceptable fit (CFI = 0.975; RMSEA = 0.078; SRMR = 0.061), but item 16 presented a very low factor loading (0.050; *p* = 0.231). After removing it, the 15-item model showed improved fit (CFI = 0.980; RMSEA = 0.055; SRMR = 0.057) and all factor loadings exceeded 0.500, except for item 7 (0.084; *p* = 0.033), which was retained for theoretical relevance. Internal consistency was high (α = 0.87; ω = 0.88; CR = 0.89; AVE = 0.62).

**Conclusion:**

The 15-item TPS-E showed adequate psychometric properties in Ecuadorian university students, being a reliable and valid instrument for assessing academic procrastination in this context. Its use in research and intervention is recommended, and future adaptations should explore convergent validity and potential cultural differences affecting specific items.

## Introduction

Procrastination is a behavioral phenomenon characterized by the habitual postponement of important tasks until the last possible moment, affecting individuals across a wide range of contexts, particularly in occupational and academic domains (Ferrari & Roster, 2018; Infante Borinaga & Echeazarra Escuero, 2019; Muliani et al., 2020). The literature distinguishes between two main types: decisional procrastination, involving delays in making choices (Ferrari, 2001), and behavioral procrastination, involving delays in task execution (Zarzycka et al., 2019). In academic settings, procrastination often manifests through behaviors such as postponing exam preparation or assignment completion, which can lead to discomfort and distress (Milgram et al., 1998). Research by Özer (2011) indicates that more than 70% of university students procrastinate on academic tasks at least occasionally—a behavior linked to lower academic performance and elevated levels of stress and anxiety (Kim & Seo, 2015).

According to Self-Regulation Theory, procrastination reflects an inability to effectively manage one’s internal resources (Park & Sperling, 2012; Van Eerde & Klingsieck, 2018). Similarly, Zhou (2022) associates academic procrastination with self-regulation difficulties, whereby individuals struggle to control critical factors such as motivation, attention, and time management, ultimately failing to meet academic deadlines (Nuñez et al., 2010; Nuñez Alonso, 2006; Nuñez Alonso et al., 2005; Stover et al., 2015).

These deficits in self-regulation may present as: (a) time management problems, where important tasks are deferred in favor of less significant but more immediate activities; (b) difficulties in emotional regulation, often driven by anxiety and fear of failure, which undermine focus and long-term motivation; and (c) decision-making challenges, in which individuals have trouble determining when and how to begin a task, thereby perpetuating the cycle of procrastination (Zhou et al., 2022).In this context, the Tuckman Academic Procrastination Scale (TPS) serves as a valid and reliable psychometric tool to assess tendencies toward time-wasting, intentional delays, and procrastination in task completion (Tuckman, 1990). Originally, the TPS consisted of 72 items measuring: a) general self-description of procrastination tendencies, b) difficulties in tackling unpleasant tasks, leading to their avoidance, and c) a tendency to blame others for personal shortcomings. The TPS was first tested on a student sample and subjected to factor analysis, resulting in a two-dimensional, 35-item version. This version was later administered to a new sample, leading to the development of a 16-item version that showed a single factor with an α of .86 (Tuckman, 1991).

This 16-item version was adapted and validated in Canada by Timothy A. Pychyl (1998) to more accurately reflect procrastination behaviors in the educational context (Pychyl 2000). A Spanish version of the scale, applied to university students in Buenos Aires by Furlan et al., (2012a, b), was based on a 15-item adaptation due to one item having a factor loading lower than .40. The internal consistency of the Spanish version (ATPS) was acceptable (α = .87), exceeding the standard threshold of .70 for typical behavior scales. This version included both direct (e.g., “I unnecessarily delay finishing work, even if it is important”) and reverse-coded questions (e.g., “I start working immediately, even on tasks I find unpleasant”). A Likert-type scale was used, with responses ranging from 1 (never) to 5 (always). Both the original (Tuckman, 1990) and adapted versions (Furlan et al., 2012a, b) reported a Cronbach's α of .87. The translation and cross-cultural adaptation process followed the International Testing Commission's guidelines to ensure equivalence between the original and translated items (Muñiz et al., 2013).

Procrastination, particularly academic procrastination, has long been recognized as a behavior associated with significant challenges in self-regulation. Research consistently supports the idea that personality traits play a critical role in the development of academic procrastination (Johnson & Bloom, 1995; Lee et al., 2005; Ozer et al., 2009; Senécal et al., 2003). Academic procrastination is typically characterized by a predominant tendency to delay essential academic tasks, a behavior frequently linked to anxiety and discomfort (Guerra and Jorquera, 2021; Harpell and Andrews, 2012; Heredia et al., 2008; Mowbray et al., 2015). For Moonaghi and Beydokhti (2020), procrastination is a prevalent phenomenon, particularly among university students. It often leads them to prioritize less important tasks, thereby avoiding responsibility and commitment, which ultimately negatively impacts academic performance (DiStefano and Motl, 2006; Rojas-Torres et al., 2023; Estremadoiro Parada & Schulmeyer, 2020).

Solomon and Rothblum (1984) define academic procrastination as the persistent delay of academic tasks due to anxiety, resulting in discomfort and avoidance patterns when students face tasks perceived as high-cost or when the chances of achieving high satisfaction with their performance are considered slim (Bui, 2007). Tuckman (1990) suggests that academic procrastination reflects a lack of self-regulation, as seen in students' tendencies to avoid tasks under their control and responsibility, displaying little effort in task completion. This behavior pattern is often accompanied by subjective discomfort, which represents a real problem of self-regulation on cognitive, affective, and behavioral levels (Steel & Klingsieck, 2016).

From a psychological perspective, Steel and Klingsieck (2016) categorize procrastination into four dimensions: 1) Individual differences, where procrastination is seen as a trait-like personality construct (e.g., conscientiousness, neuroticism, perfectionism, and self-esteem); 2) Motivational perspective, which addresses procrastination as a failure in both motivation and volition (e.g., self-determination and locus of control); 3) Subjective well-being perspective, which is related to self-regulation issues, such as depression and anxiety, and emotional aspects like task aversion (e.g., boredom during task execution); and 4) Social-demographic perspective, which considers demographic factors (e.g., social models and gender roles) influencing procrastination tendencies.

In academic contexts, procrastination is especially common, as most academic tasks are time-sensitive, yet students often prefer to engage in non-academic activities (Tuckman, 2005). Despite its widespread presence in educational settings, procrastination remains underexplored in academic research (Díaz Morales, 2019). However, procrastination is more than just a behavioral issue; it is closely tied to cognitive factors such as biased beliefs about work and affective components like emotional states related to task completion (Chun Chu & Choi, 2005; Klingsieck, 2013). Procrastination may also be influenced by perceived self-competence (Haghbin et al., 2012), low self-control (Uzun et al., 2020), fear of failure (Zhang et al., 2018), depression (Kınık & Odaci, 2020), low self-esteem (Hajloo, 2014), and anxiety (Hodapp, 1991; Hoddap, 1995; Hoddap and Benson, 1997; Hoddap et al., 1982; Hoddap et al., v 2011; Keith et al., 2003; Mowbray et al., 2014; Spada, 2006). Previous studies have found negative correlations between academic procrastination and important variables, such as academic satisfaction (Balkis & Duru, 2017), intentions to continue studying (Bäulke et al., 2018), effective learning strategies (Howell & Watson, 2007), academic commitment (Aspée et al., 2021), and academic performance (Goroshit, 2018; Kim & Seo, 2015; Kim et al., 2017).

This research is particularly important for understanding the underlying psychological mechanisms of academic procrastination, as well as its practical implications for students, educators, and institutions. Despite the considerable body of literature on academic procrastination, there remain gaps in understanding the specific factors that contribute to this behavior and how they interact. For instance, while various personality traits and cognitive factors have been identified, less is known about the role of cultural and educational contexts in shaping procrastination tendencies. Moreover, existing tools for measuring procrastination, such as the Tuckman Academic Procrastination Scale (TPS), require further validation to ensure they are both reliable and culturally appropriate for diverse student populations.

This study aims to fill these gaps by validating the TPS within a specific cultural and academic context, enhancing its relevance for research and intervention. By focusing on the psychometric properties of the scale, this study will provide valuable insights into the nature of procrastination behaviors among university students and contribute to the development of more effective strategies for addressing procrastination in educational settings. Ultimately, this research seeks to improve our understanding of academic procrastination and support the development of interventions that can help students better manage their academic responsibilities and enhance their overall well-being (Melnick and Gable, 1990; Mowbray et al., 2014).

Several studies have evaluated the psychometric properties of the Tuckman Procrastination Scale (TPS) within academic settings. Furlan et al., (2010) conducted a direct Spanish translation of the 35 original items and tested it with a sample of 227 Psychology students at the National University of Córdoba, Argentina. Through exploratory factor analysis (EFA), the scale was reduced to 15 items with factor loadings below 0.40. These items were grouped into a single factor explaining 33.2% of the variance, demonstrating high internal consistency (α = 0.87).

Subsequently, the adapted version of the scale underwent confirmatory factor analysis (CFA) to determine its internal structure. The fit indices supported a unidimensional model (RMSEA = 0.045, CFI = 0.99, GFI = 0.98, ACI = CMIN/DF = 2.655) with adequate internal consistency (α = 0.94). Statistically significant negative correlations were found between TPS scores and academic performance (*r* = -0.217, *p* < 0.001) as well as between TPS scores and test anxiety (*r* = 0.430, *p* < 0.000).

Similarly, Galindo Contreras and Olivas Ugarte (2022) worked with a sample of 429 Peruvian students selected through non-probabilistic convenience sampling. The initial CFA for the 15-item unidimensional model did not provide a good fit, leading to the removal of five items. After adjustments, the model fit indices indicated a good fit (X^2^/df = 1.681, CFI = 0.992, TLI = 0.990, RMSEA = 0.040, SRMR = 0.030). Reliability was confirmed with both Cronbach’s alpha (α = 0.85) and omega coefficient (ω = 0.86).

In addition, Alegre Bravo and Benavente Dongo (2020) analyzed the psychometric properties of the TPS with a sample of 764 students from a private university in Lima, Peru. An exploratory factor analysis (EFA) revealed a unidimensional structure underlying the original 15 items. However, three items had low factor loadings (< 0.32), so a 12-item reduced version was proposed, which showed better fit indices: RMSEA = 0.056 (< 0.60), CFI = 0.979 (> 0.95), and GFI = 0.986 (> 0.95). Both Cronbach’s alpha (α = 0.859) and omega coefficient (ω = 0.860) reflected optimal internal consistency.

Despite these international efforts, there is no empirical evidence regarding the validity and reliability of the TPS in the Ecuadorian context. Given the significant impact of academic procrastination in higher education and the need for tools to assess, diagnose, and guide research, this study aims to address this gap. The objective is to perform a psychometric analysis of the 16-item TPS in Ecuador, providing empirical evidence of its suitability for use in this context.

Considering the results from prior studies on the validity and reliability of the Tuckman Procrastination Scale (TPS) adaptations, this study aims to assess the applicability of the Ecuadorian adaptation of the TPS, focusing on its psychometric properties within the Ecuadorian population. Given the absence of robust, culturally appropriate tools for evaluating procrastination tendencies in Ecuador, this research seeks to establish the reliability, validity, and unidimensionality of the scale. Specifically, the goal is to validate the TPS-E as a reliable and valid instrument for measuring academic procrastination tendencies, ensuring that it is consistent with the theoretical framework underlying the scale. Hypothesis 1 posits that the Ecuadorian adaptation of the Tuckman Academic Procrastination Scale (TPS-E) will demonstrate adequate psychometric properties, including a good model fit. We expect the Comparative Fit Index (CFI) to reach values of 0.95 or higher, indicating a good fit between the data and the proposed model. Furthermore, we hypothesize that Cronbach’s Alpha and McDonald’s Omega values will exceed 0.80, reflecting strong internal consistency, similar to the values reported in previous cultural adaptations. Additionally, we anticipate that item 16, if deemed theoretically relevant, will contribute to the construct in subsequent analyses after considering possible cultural or translation-related issues.

## Methods

### Participants

A non-probabilistic, consented and anonymous sample of 1007 (*n* = 403 women, 39.9%) participants with an age average of 21.88 years (*SD* = 3.69) took part on the study. Subjects belong to the following Ecuadorian Private University Faculties: Social Sciences and Humanities (*n* = 285, 28.3%), Science and Technology (*n* = 399, 39.6%), Administration and Economics (*n* = 158, 15.7%), Life Sciences (*n* = 113, 11.2%) and Education (*n* = 52, 5.2%).

### Instrument

#### The Tuckman Academic Procrastination Scale (TPS)

As previously described, Tuckman (1990) developed the Tuckman Procrastination Scale (TPS), a psychometric instrument designed to assess the tendency to delay tasks unnecessarily, grounded in the cognitive and rational–emotive theoretical model proposed by Ellis and Knaus (1977). This model conceptualizes procrastination as a self-regulation failure influenced by maladaptive cognitions, irrational beliefs, and avoidance-oriented behaviors (Ellis and Knaus, 2002). The TPS was subsequently adapted for the Argentine context by Furlan et al., (2012a, b) following rigorous linguistic and cultural adaptation procedures to ensure conceptual equivalence with the original version.

The TPS is specifically aimed at measuring procrastination tendencies in university students and consists of 16 items rated on a five-point Likert scale, ranging from 1 (never happens to me) to 5 (always happens to me). The items capture behaviors related to intentional delays, avoidance of unpleasant tasks, and inefficient time management. Psychometric evaluations have demonstrated strong measurement properties, with a Comparative Fit Index (CFI) of 0.99, indicating excellent model fit, and reliability coefficients Cronbach’s alpha and McDonald’s omega, for each factor exceeding the threshold of 0.80, reflecting high internal consistency (Tuckman, 1990). These results confirm the instrument’s suitability for both research and applied educational settings, providing a reliable means of identifying procrastination patterns that can negatively impact academic performance and student well-being.

### Procedure

Before the study began, ethical approval was obtained from the relevant university or institutional review board to ensure compliance with ethical guidelines. All participants were provided with a clear and detailed informed consent form, explaining the purpose of the study, the procedures involved, any potential risks, and the confidentiality of their data. Participants were required to sign the informed consent form voluntarily before being included in the study.

The confidentiality of participants was maintained throughout the study. No personally identifiable information was collected or disclosed, and all responses were kept confidential. Data were anonymized and securely stored to prevent unauthorized access, ensuring that participants could be assured their involvement would not impact their academic standing or any other personal interests.

Initially, a group of judges consisting of university professors and researchers conducted a linguistic review of the Furlan et al., (2012a, b) Spanish-adapted version of the instrument before applying it to the sample during a pilot study. This review process included evaluations of wording, coherence, and relevance (Elousa, 2003) for each item to ensure that the instrument was clear and suitable for the Ecuadorian context. It was determined that no items or factors needed to be eliminated, and no additional content validation process was necessary, as the instrument showed consistency in its format and structure.

The instrument was then applied to an initial group of ten students, who independently assessed the linguistic clarity of the items by providing feedback on any words or phrases considered confusing or difficult to understand. Their feedback was essential in refining the instrument's readability, ensuring that it was fully understandable to the entire study population.

Finally, Table 1 presents the final Ecuadorian-adapted TPS items alongside the original Furlan et al., (2012a, b) version. This adaptation process ensured that the instrument was both linguistically and culturally appropriate, thus increasing the validity and reliability of the results not only during the pilot study but also for future research in Ecuador.

**Table 1:** Argentinean (ATPS) and Ecuadorian (TPS-E) Tuckman Academic Procrastination Scale item-linguistic understanding comparison

ATPS Items (*Argentinean*)	TPS-E Items (*Ecuadorian*)	TPS-E Items (*Ecuadorian*) in English
(Furlan et al., 2012a, b)		
1. Retraso innecesariamente la finalización de los trabajos, incluso cuando son importantes	1. Retraso innecesariamente la finalización de los trabajos, incluso cuando son importantes	1. I unnecessarily delay finishing tasks, even when they’re important
2. Pospongo comenzar con cosas que no me gustan hacer	**2. Pospongo el inicio de cosas que no me gustan hacer**	2. I put off starting things I don’t like to do
3. Cuando tengo una fecha límite, espero hasta el último minuto	3. Cuando tengo una fecha límite, espero hasta el último minuto	3. When I have a deadline, I wait until the last minute
4. Retraso la toma de decisiones difíciles	4. Retraso la toma de decisiones difíciles	4. I postpone making difficult decisions
5. Sigo postergando mejorar mis hábitos de trabajo	5. Sigo postergando mejorar mis hábitos de trabajo	5. I keep putting off improving my work habits
6. Logro encontrar una excusa para no hacer algo	6. Logro encontrar una excusa para no hacer algo	6. I always manage to find an excuse not to do something
7. Dedico el tiempo necesario incluso a tareas aburridas, como estudiar	7. Dedico el tiempo necesario incluso a tareas aburridas, como estudiar	7. I devote the necessary time even to boring tasks, like studying
8. Soy un perdedor de tiempo incorregible	**8. Pierdo el tiempo de manera incorregible**	8. I waste time incorrigibly
9. Yo suelo perder el tiempo, pero parece que no puedo hacer nada al respecto	9. Yo suelo perder el tiempo, pero parece que no puedo hacer nada al respecto	9. I usually waste time, but it seems like I can’t do anything about it
10. Cuando algo es demasiado difícil de abordar, considero que es mejor posponerlo	10. Cuando algo es demasiado difícil de abordar, considero que es mejor posponerlo	10. When something feels too hard to tackle, I think it’s better to postpone it
11. Me prometo a mí mismo que haré algo y luego no quiero hacer las cosas	**11. Me comprometo en hacer algo y luego demoro en hacerlo**	11. I commit to doing something and then delay doing it
12. Siempre que hago un plan de acción, lo sigo	12. Siempre que hago un plan de acción, lo sigo	12. Whenever I make an action plan, I stick to it
13. Aunque me odie a mí mismo si no empiezo, igual no me hace seguir adelante	**13. Aunque me odie por no empezar, no me nace hacerlo**	13. Even if I hate myself for not starting, I just can’t get myself to do it
14. Siempre termino los trabajos importantes con tiempo de sobra	14. Siempre termino los trabajos importantes con tiempo de sobra	14. I always finish important work with plenty of time to spare
15. Me quedo estancado como bloqueado, aunque sé lo importante que es empezar	15. Me quedo atascado como bloqueado, aunque sé lo importante que es empezar	15. I get stuck, almost blocked, even though I know how important it is to get started
16. Dejar algo para mañana no es mi forma de actuar	16. Dejar algo para mañana no es mi forma de actuar	16. Putting things off until tomorrow is not my way of doing things

### Data Analysis

Instrument application data underwent Confirmatory Factor Analysis (CFA) through Diagonal Weighted Least Squares (DWLS) estimator via polychoric correlation matrix with the R-Studio software (Software Libre GNU, 2024). Analysis used the lavaan package DWLS estimator (Rosseel, 2012). Expected indices included a ≥ 0.05 significance χ2, a Robust ≥ 0.95 Comparative Fit Index, the standardized ≤ 0.08 residual square root (SRMR) and a ≤ 0.07 [90% CI 0.00 and 0.08] mean approximation error (RMSEA).

The CFA structure relationships were illustrated through Onyx (von Oertzen et al., 2015). In addition, a reliability analysis was performed by using both Cronbach's Alpha (α) and McDonald's Omega (ω) coefficients (Revelle & Zinbarg, 2009). Confirmatory factor analysis required several samples equivalent to 10 times the number of observed variables (Omer Mustafa, 2019), therefore, our sample (*n* = 1007) is considered sufficient, since it exceeds the large samples threshold (Müller Gilchrist et al., 2013).

## Results

The confirmatory factor analysis (CFA) of the TPS-E assessed its factorial structure validity based on a theoretical model, which posits that the absence of self-regulation is reflected in the subject’s tendency to avoid activities requiring self-control. This avoidance manifests as putting minimal effort into completing tasks and frequently evading situations that involve responsibility and commitment.

To verify the latent structure factors of the Academic Procrastination Model, the multivariate data distribution properties were examined. The results indicated a lack of compliance with the assumption of multivariate normality, as confirmed by the Mardia test. Additionally, the *R lavaan* package was used to evaluate the data-model fit using three indicators: the Comparative Fit Index (CFI), with values close to or higher than 0.95; the Root Mean Square Error of Approximation (RMSEA), with values close to or lower than 0.08; and the Standardized Root Mean Square Residual (SRMR), with an upper confidence interval limit below 0.08.

**Fig. 1:**
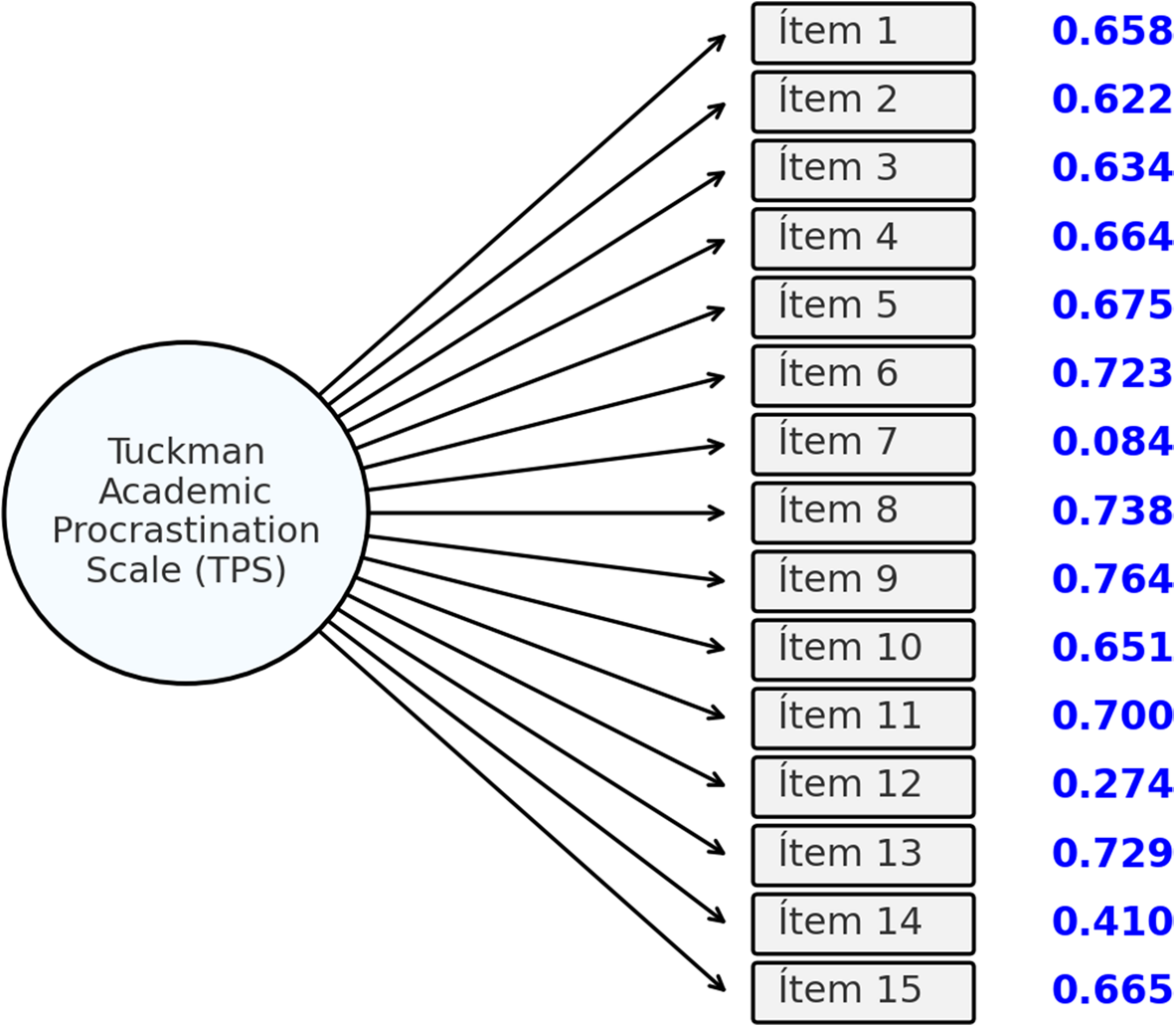
Academic procrastination model confirmatory factor analysis diagram

The model assumes the scale is unidimensional, strictly aligned with its purpose (Tuckman, 1991). To validate the construct, the observed indices showed a good fit, as illustrated in Fig. 1. The results of the Diagonal Weighted Least Squares (DWLS) CFA were as follows: CMIN or χ^2^ (104) = 746.347, *p* < 0.001; Robust CFI = 0.975, RMSEA = 0.078 (confidence interval range 0.073–0.084), and SRMR = 0.061.

The RMSEA result (> 0.08) suggested a suboptimal upper limit for the hypothesized unidimensional model. Upon reviewing the factor loadings for each item, it was found that item 16 had a non-significant contribution, negatively affecting the test's reliability. Consequently, a second model was proposed that omitted item 16. This revised model yielded a better fit, with all CFA indicators showing significant improvements: CMIN or χ^2^ (90) = 638.648, *p* < 0.001; Robust CFI = 0.980, RMSEA = 0.055 (confidence interval range 0.051–0.059), and SRMR = 0.057.

As noted, model 1 revealed a low factor loading (0.050) for item 16 and a non-significant contribution (z = 1.197; *p* = 0.231), leading to its omission. In contrast, model 2 showed factor loadings greater than 0.500 for most items (see Table 2). However, item 7 ("I dedicate the necessary time even to boring tasks") had a low factor loading of 0.084, but its contribution remained significant (z = 2.13; *p* = 0.033), so it was retained in the model.

**Table 2:** Factor loadings for the TPS-E Estimated and standardized observed variables

	Estimate	Std.Err	Z-value	*P* ( >|z|)	Std.lv	Std.all
Ítem 1	1.000				0.607	0.658
Ítem 2	0.947	0.045	21.044	0.000	0.574	0.622
Ítem 3	0.988	0.049	20.007	0.000	0.599	0.634
Ítem 4	1.014	0.051	19.925	0.000	0.615	0.664
Ítem 5	1.045	0.053	19.656	0.000	0.634	0.675
Ítem 6	1.117	0.051	22.040	0.000	0.678	0.723
Ítem 7	0.118	0.056	2.100	0.036	0.072	0.084
Ítem 8	1.137	0.050	22.596	0.000	0.689	0.738
Ítem 9	1.162	0.052	22.302	0.000	0.705	0.764
Ítem 10	0.976	0.051	19.070	0.000	0.592	0.651
Ítem 11	1.072	0.051	20.967	0.000	0.650	0.700
Ítem 12	0.371	0.051	7.273	0.000	0.225	0.274
Ítem 13	1.167	0.054	21.554	0.000	0.708	0.729
Ítem 14	0.575	0.051	11.233	0.000	0.349	0.410
Ítem 15	0.976	0.049	19.971	0.000	0.592	0.665

Given that previous literature has proposed an established theoretical model, the present study opted for a direct Confirmatory Factor Analysis (CFA) approach, as opposed to the conventional sequential method. In the sequential approach, it is typical to first conduct an Exploratory Factor Analysis (EFA) to identify the factor structure before performing a CFA. However, our decision was influenced by the strength of prior studies that suggest the TPS has a unidimensional structure characterized by the absence of self-regulation within the framework of academic procrastination (Tuckman, 1991). This theoretical foundation supports the choice of CFA, as it focuses on confirming the predefined factor structure rather than exploring new, potentially uncertain patterns.

Moreover, Bollen (1989) underscores the importance of using CFA over EFA when working with pre-existing theories and well-defined models. This perspective aligns with the goals of the current study, as we sought to validate the hypothesized model, not to explore or redefine it. By choosing CFA, we were able to specifically test the validity of the proposed theoretical model within the context of academic procrastination, aligning with the principles set forth in previous research. Thus, this decision reflects a methodological approach that emphasizes model testing and theory confirmation, rather than exploration.

For the internal consistency analysis of the TPS-E, we employed two widely recognized reliability coefficients: Cronbach’s Alpha (α) and McDonald's Omega (ω). These measures are commonly used to assess the reliability and consistency of psychological scales and questionnaires. The results revealed that the reliability levels were optimal for nearly all indicators, with coefficient values approaching 0.900 for model 2. This suggests that the scale items demonstrate a high degree of internal consistency, reinforcing the model’s validity.

However, in analyzing the factor loadings, it was found that item 16 contributed non-significantly to the model, which negatively affected the overall test reliability. Consequently, a second model was proposed that omitted item 16. It is important to emphasize that the questionnaires used in this study respond to a theoretical model. Data analysis should confirm the theoretical model, not the other way around. Therefore, if item 16 is theoretically part of the construct, and the data analysis does not support its inclusion, especially if this is not the case in other cultural adaptations or in the original questionnaire, it should not be simply deleted. Instead, the potential reasons for this discrepancy should be explored. Possible causes include cultural differences that may affect how respondents interpret the item, or issues such as an inaccurate translation. In fact, the formulation of item 16 includes a double negative, which could make it difficult for some respondents to correctly interpret the question and select an appropriate response on a Likert scale.

Despite the concerns regarding item 16, the overall reliability of the model, calculated with all items included, suggested that retaining this item did not negatively impact the model’s consistency. Consequently, despite its low factor loading, the comprehensive reliability analysis provided sufficient justification for keeping all other items in the final model.

In conclusion, the decision to bypass the typical EFA phase and proceed directly with CFA was driven by the strong theoretical framework supporting the unidimensional structure of the TPS, as well as the methodological recommendations of prior research. The results of the internal consistency analysis further supported the robustness of the model, ensuring that the proposed structure remains valid and reliable for assessing academic procrastination.

## Discussion

The purpose of this study was to evaluate the Ecuadorian adaptation of the Tuckman Academic Procrastination Scale (TPS-E) in a sample of 1,007 university students from Ecuador. The results provide strong evidence of the psychometric properties of the adapted scale, establishing its reliability and validity for use in the Ecuadorian context. The findings underscore the significance of using culturally adapted instruments in psychological research and practice, contributing to a more accurate assessment of academic procrastination among Ecuadorian students.

While cultural and linguistic differences exist between Ecuador and other Latin American countries, the TPS-E demonstrated significant psychometric strengths that align with previous validations of procrastination measures in diverse cultural environments (Ringeisen et al., 2010; Rohrmann et al., 2010). These results highlight the importance of cross-cultural adaptability in psychological measurement tools. The effectiveness of the TPS-E in Ecuador reinforces the need for culturally sensitive instruments, ensuring that they are both relevant and accurate across different sociocultural contexts (Schriesheim et al., 1991; Uzun et al., 2020).

The process of adapting the TPS-E involved a thorough linguistic review and pilot testing, as recommended by Núñez et al. (2006), with the input of expert judges and university students. This iterative approach, which considered local linguistic peculiarities, reflects best practices in the adaptation of psychometric instruments (Van de Vijver and Tanzer, 2004). The resulting scale effectively captures the nuances of procrastination in the Ecuadorian university student population, offering a tool that is contextually appropriate and relevant.

The confirmatory factor analysis (CFA) revealed that Model 1 required the removal of item 16 due to a low factor loading (0.050) and non-significant contribution (z = 1.197, *p* = 0.231). On the other hand, Model 2, which retained all items except item 7, showed a satisfactory factor structure and a significant contribution (z = 2.13, *p* = 0.033). This refinement of the model demonstrates the delicate balance between statistical rigor and theoretical consistency, as outlined by Kline (2015) and Hair et al. (2018). The internal consistency of the revised 15-item scale (α = 0.87) and the unidimensional structure (α = 0.94) provide strong support for the reliability of the scale. These results mirror findings from previous studies on academic procrastination, reinforcing the scale’s applicability to similar academic contexts (Senécal et al., 2003; Steel & Klingsieck, 2016).

In terms of model fit, the indices CMIN/χ^2^ (90) = 638.648, *p* < 0.001, CFI = 0.980, RMSEA = 0.055, SRMR = 0.057 demonstrate strong support for the psychometric robustness of the TPS-E. These values are consistent with those observed in other studies evaluating procrastination and self-regulation measures, such as Van Eerde & Klingsieck (2018) and Zhou et al., (2022), providing further evidence of the scale’s validity in academic settings.

The validation of the TPS-E is also aligned with Vallerand’s (1997) hierarchical model of motivation, which posits that procrastination is influenced by both intrinsic and extrinsic motivational factors. The results underscore the importance of considering motivational constructs in understanding procrastination behaviors (Côté and Levine, 2000; Deci and Ryan, 1985; Deci and Ryan, 2004; Grouzet et al., 2004; Vallerand et al., 1998; Vallerand et al., 1992). In this sense, the scale provides valuable insights for both practitioners and theorists in the field of educational psychology, offering a reliable tool to assess procrastination tendencies and inform intervention strategies (Barkoukis et al., 2008; Barnette, 2000; Fairchild et al., 2005).

When comparing these results to those of Alegre Bravo and Benavente Dongo (2020) and Galindo Contreras and Olivas Ugarte (2022), who excluded multiple items due to low factor loadings, our study presents a more refined version of the TPS-E with higher internal consistency (α = 0.87) and stronger fit indices, validating its use among Ecuadorian university students. The robustness of these findings emphasizes the potential of the TPS-E to inform interventions aimed at reducing academic procrastination, which is a pervasive issue affecting students' academic performance and well-being (Spada et al., 2006; Tuckman, 1991, 2005).

The application of advanced statistical techniques, such as structural equation modeling (SEM) using the *lavaan package in R* (Jöreskog and Sörbom, 1993; Jöreskog and Sörbom, 1996; Rosseel, 2012), further enhances the reliability and validity of the TPS-E. The inclusion of McDonald’s omega (ω = 0.88), along with Cronbach’s alpha, provides a more nuanced understanding of reliability, while the composite reliability (CR = 0.89) and average variance extracted (AVE = 0.62) ensure robust validity estimates (Hair et al. 2018; Stover et al., 2012). These methodological rigor increases the scale’s precision and confidence in its use for both research and practical applications in academic settings.

Despite the promising findings, the study has limitations, such as the lack of participation from senior university students, which may affect the generalizability of the results. Future research should aim to incorporate a more diverse sample, including students from various academic levels and geographic regions in Ecuador, to enhance the external validity of the findings. This would allow for the exploration of potential variations in procrastination behaviors across different subgroups, as suggested by Ringeisen et al., (2010) and Van de Vijver and Tanzer (2004). Additionally, incorporating behavioral measures of procrastination (Solomon & Rothblum, 1984) alongside self-reported data could provide a more comprehensive assessment of procrastination tendencies, offering further insights into the factors that contribute to this phenomenon.

Future research could also benefit from longitudinal studies to investigate the developmental trajectories of procrastination and its long-term effects on academic outcomes. Integrating findings from studies on test-enhanced learning (Roediger & Karpicke, 2006) and memory retention could inform interventions designed to reduce procrastination and improve academic achievement, providing a basis for evidence-based interventions in university settings.

In conclusion, the TPS-E has demonstrated strong psychometric properties, including reliability and validity, making it a valuable tool for assessing academic procrastination in Ecuadorian university students. The study highlights the importance of culturally sensitive adaptations of psychological instruments and provides a foundation for future research on procrastination in Latin American academic contexts. Practically, the TPS-E can inform interventions aimed at improving students’ academic behaviors, offering valuable insights for educators and psychologists working to address procrastination and its negative impact on student performance and well-being.

In the context of the Ecuadorian adaptation of the Tuckman Procrastination Scale (TPS-E), this study demonstrates the psychometric strength of the scale, confirming its reliability and validity for assessing academic procrastination tendencies. Despite its overall success, some items, including item 16, required revision or elimination to improve the instrument’s fit and relevance. Specifically, the removal of item 16 was based on its low factor loading (0.050) and non-significant contribution to the model (z = 1.197, *p* = 0.231). The elimination of this item, although necessary, raises important considerations regarding its impact on the scale’s validity. In particular, it is crucial to justify the removal of an item in the discussion, explaining how it did not align with the underlying theoretical structure or how it failed to reflect cultural nuances in the Ecuadorian context. Such justifications are important for ensuring that the decision to exclude an item is transparent and consistent with both statistical criteria and theoretical frameworks.

The decision to exclude item 16 reflects a broader challenge in cross-cultural adaptations of psychological instruments, where items may not resonate similarly across diverse cultural contexts. It is critical to acknowledge that, while item 16 was theoretically relevant, its psychometric performance in the Ecuadorian sample indicated that it did not sufficiently contribute to the construct of procrastination as measured by the TPS-E. This provides an opportunity for future research to explore potential cultural or translation-related issues that might influence the instrument’s performance and suggests that additional psychometric evaluations are needed to refine and optimize the scale.

A limitation of this study, which was not sufficiently addressed in the original manuscript, is the lack of discussion on the implications of eliminating item 16. The deletion of an item can impact both the internal consistency and the construct validity of the instrument. This requires careful reflection in the discussion to ensure that such eliminations are grounded in sound theoretical and statistical reasoning. Moreover, the absence of an exploration into other types of validity, such as convergent validity, further limits the understanding of the scale's robustness. Convergent validity, which assesses the degree to which the scale correlates with other measures of procrastination or related constructs, is particularly important when eliminating items. Future studies should explore this form of validity to better understand the overall construct measured by the TPS-E and to provide a more comprehensive evaluation of the scale's psychometric properties.

Building on the findings from previous studies on the Tuckman Procrastination Scale (TPS) and its adaptations, this study confirms that the Ecuadorian version of the scale demonstrates adequate psychometric properties, including good model fit and internal consistency. However, future research should address the limitations noted in this study, such as the justification for item eliminations and the exploration of other forms of validity. Specifically, investigating the relationship between procrastination tendencies and other psychological constructs, as well as performing a multigroup analysis to ensure the scale’s applicability across different student subgroups, will further validate the scale’s cross-cultural utility. Furthermore, researchers should consider alternative models that could better account for low-loading items, ensuring that the TPS-E remains a robust and adaptable tool for assessing procrastination in various academic and cultural settings.

## Conclusions

The TPS-E demonstrated strong psychometric performance, confirming its reliability and validity for assessing academic procrastination among Ecuadorian university students. The confirmatory factor analysis supported a unidimensional structure, and the adapted version maintained conceptual coherence with the original theoretical framework proposed by Tuckman. The removal of one item due to its low and non-significant contribution improved the model’s fit, underscoring the importance of balancing statistical evidence with theoretical relevance in cross-cultural adaptations. The decision to retain another item with a lower factor loading was supported by its theoretical significance, illustrating that psychometric refinement must consider both empirical evidence and conceptual alignment. These findings highlight the value of the TPS-E as a useful tool for educational research and practice, capable of guiding psychoeducational interventions, monitoring changes in procrastination behaviors, and supporting strategies to reduce the negative academic and psychological consequences of this phenomenon. By providing a culturally adapted and psychometrically sound instrument, this study enhances assessment accuracy and strengthens the capacity to deliver targeted educational support in Ecuadorian universities.

Although the results confirm the adequacy of the TPS-E, several limitations should be considered when interpreting the findings. The non-probabilistic sample from a single private university may limit the generalizability of the results to other academic contexts or institutions in the country. The exclusive use of self-report measures could be influenced by social desirability bias or subjective perceptions of procrastination. Complementary analyses, such as convergent validity or measurement invariance, were not conducted, which would provide a more comprehensive understanding of the instrument’s performance across different subgroups. Furthermore, the cross-sectional design does not allow conclusions about the temporal stability of the scale or changes in procrastination behaviors over time. Addressing these aspects in future research would strengthen the evidence for the TPS-E’s applicability in diverse educational settings.

## Data Availability

The data used in this study are stored and managed by the corresponding author, who can be contacted for any inquiries. Due to ethical considerations, the data are not publicly available, as the researchers are committed to protecting participant privacy and ensuring that the data are used solely for academic purposes. The ethics committee was approved by COBIAS at the University of Cuenca (Code: 2022-011EO).

## Abbreviations

AP: Academic procrastination
EUS: Ecuadorian University Students
CFA: Confirmatory factor analysis
ICA: Internal consistency analysis
